# Rehabilitative management of an infant with Pelizaeus–Merzbacher disease: A case report

**DOI:** 10.1097/MD.0000000000020110

**Published:** 2020-05-29

**Authors:** Yun-Chol Jang, Bo-ra Mun, In Sung Choi, Min-Keun Song

**Affiliations:** Department of Physical & Rehabilitation Medicine, Chonnam National University Medical School & Hospital, Gwangju City, Republic of Korea.

**Keywords:** Pelizaeus–Merzbacher disease, rehabilitation

## Abstract

**Rationale:**

Pelizaeus–Merzbacher disease (PMD) is an X-linked recessive trait and a rare disease characterized by abnormal myelin formation in the central nervous system. Since Pelizaeus and Merzbacher reported the pathology of PMD in the 1990s most studies have examined pharmacological treatments. No studies have reported the effects of rehabilitation on patients with PMD aimed at improving their functional abilities. We report the first case of improved development after rehabilitation in a patient with Pelizaeus–Merzbacher disease.

**Patient concerns:**

A 1-month-boy developed focal seizures, nystagmus, and jerky head movements. He was brought to our outpatient clinic for rehabilitation of developmental delay at 11 months of age. He showed hypotonia, nystagmus, and developmental delay of 4 to 5 months in his gross and fine motor ability.

**Diagnoses:**

Developmental delay in a patient with PMD.

**Interventions:**

A child with PMD was hospitalized 3 times for 3 months and underwent rehabilitation to improve developmental delay. Developmental assessments were conducted before and after each admission for rehabilitation training.

**Outcomes:**

Before training, the patient was unable to maintain a sitting position. After the first and second training sessions, his gross motor ability had improved, and he could sit with a mild assist. Fine motor function also improved. Before training, the patient was able to transfer a cube from one hand to the other. After training, he could perform a pincher grasp.

**Lessons:**

Rehabilitation training can help PMD patients achieve maximal function and catch-up in their growth.

## 1 Introduction

Pelizaeus–Merzbacher disease (PMD) is a rare disease characterized by abnormal myelin formation in the central nervous system.^[1,2]^ The prevalence of PMD is 1:90,000 to 1:750,000 live births worldwide and varies demographically. The reported prevalence of PMD is 1:200,000 to 1:500,000 in the United States, 1.45 in 100,000 live births in Japan, and 0.13 in 100,000 live births in Germany.^[3–5]^

PMD is an X-linked recessive trait. Proteolipid protein (PLP1) contributes to myelination in the central nervous system (CNS). PMD is caused by a mutation in the *PLP1* gene that causes delayed CNS myelination.^[6–10]^ The most common mutation is duplication of the *PLP1* gene (60–70%). Point and null mutations can also occur.^[11]^ The symptoms of PMD vary according to the type and degree of mutation. Nystagmus, spasticity, ataxia, involuntary jerking movement, hypotonia, dysarthria, swallowing difficulty, breathing problems, and seizures are common.^[12,13]^ These symptoms are usually observed at 1 year of age.^[11]^

PMD is classified as connatal, transitional, classical, and SPG2 types based on the symptoms and mutation.^[14]^ Connatal PMD is the most severe and affected patients generally die in infancy or adolescence. The classical type is the most common type of PMD, and it mainly involves duplication of the *PLP1* gene. The initial symptoms of classical PMD are hypotonia, nystagmus, and decreased gross and fine motor function. These patients have a life expectancy of about 30 years.^[11,12]^

Since Pelizaeus and Merzbacher reported the pathology of PMD in the 1990s,^[1,2]^ most studies have examined pharmacological treatments. Although PMD patients can live 30 to 70 years, there has been little interest in PMD patients quality of life or functional ability. No studies have reported the effects of rehabilitation on patients with PMD aimed at improving their functional abilities. We report the first clinical experience of the rehabilitation of a 1-year-old boy diagnosed with PMD.

## 2 Case description

### 2.1 Patient

A boy born to a Korean father and Uzbek mother developed focal seizures, nystagmus, and jerky head movements 1 month after birth. He was brought to our outpatient clinic for rehabilitation of developmental delay at 11 months of age. The results of the Korean Developmental Screening Test for Infants & Children showed that his gross motor function was equivalent to 6 months (could change from a supine position to a prone position) and his fine motor function was equivalent to 7 months (transfer a cube from one hand to the other). He showed developmental delay of 4 to 5 months in his gross and fine motor ability.

**Figure 1:**
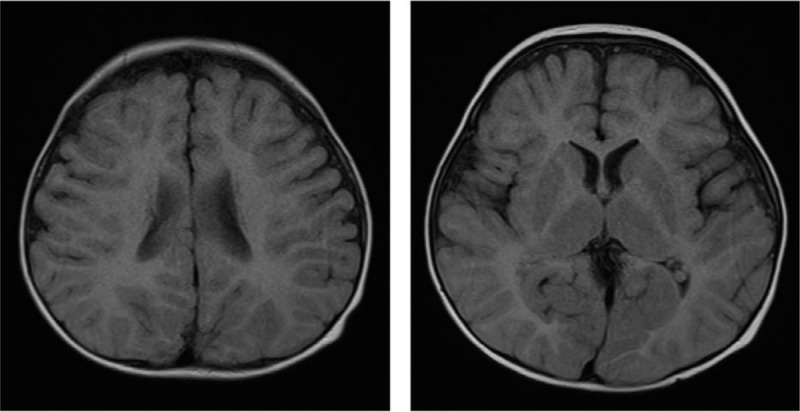
Brain MRI. MRI was performed when the patient was 11 months old. Brain MRI showed total demyelination in the bilateral white matter and dense bilateral thalami on T2W1.

Brain magnetic resonance imaging (MRI) revealed total demyelination in the bilateral white matter and dense bilateral thalami on T2 weighted images (T2W1) (Fig. 1). The patient was diagnosed with classical PMD at 1 year of age after genetic testing for the *PLP1* gene duplication (exons 1–7). There was no family history of PMD.Method

The patient was hospitalized 3 times for a rehabilitation program. He was first admitted at 12 months of age and underwent training twice a day, 5 times a week, for 1 month. Each rehabilitation session lasted 1.5 hours and consisted of neurodevelopmental and occupational therapy and functional electrical stimulation.

At the first admission, the patient still had nystagmus, jerky head movements, and ataxia of both arms and the trunk. Neck control was unstable and he could not sit independently. However, muscle strength in all extremities was fair.

To improve the nystagmus and unstable balance, the training program focused on eye tracking of slowly moving objects, maintaining head control, holding an object with the hand correctly, and maintaining a sitting position. Three weeks after discharge, he was admitted for the second time at the age of 13 months and received the same training for another month. During the period between the first discharge and second hospitalization, he was admitted to another hospital and continued to receive rehabilitation. The patient was treated for acute bronchiolitis for 1 week during the second hospitalization. Two months after the second discharge, he was admitted for the third time and underwent training for approximately 1 month. However, there was no rehabilitation treatment between the second discharge and third hospitalization. During the third training period, the patient was treated for cough variant asthma for 1 week. Except for the bronchitis and asthma, there were no other adverse events during training.

The study was approved by the Institutional Review Board of Chonnam National University Hospital (IRB No. CNUH-EXP-2019–190). The patients parents were well informed about this report and voluntarily agreed to participate. Informed consent was obtained from the patients father.Functional assessment

As mentioned, 3 rehabilitation exercises were conducted. Development was assessed before and after the rehabilitation training using manual muscle tests, range of motion, the Gross Motor Function Classification System (GMFCS), and Gross Motor Function Measure (GMFM).

## 3 Results

**Figure 2:**
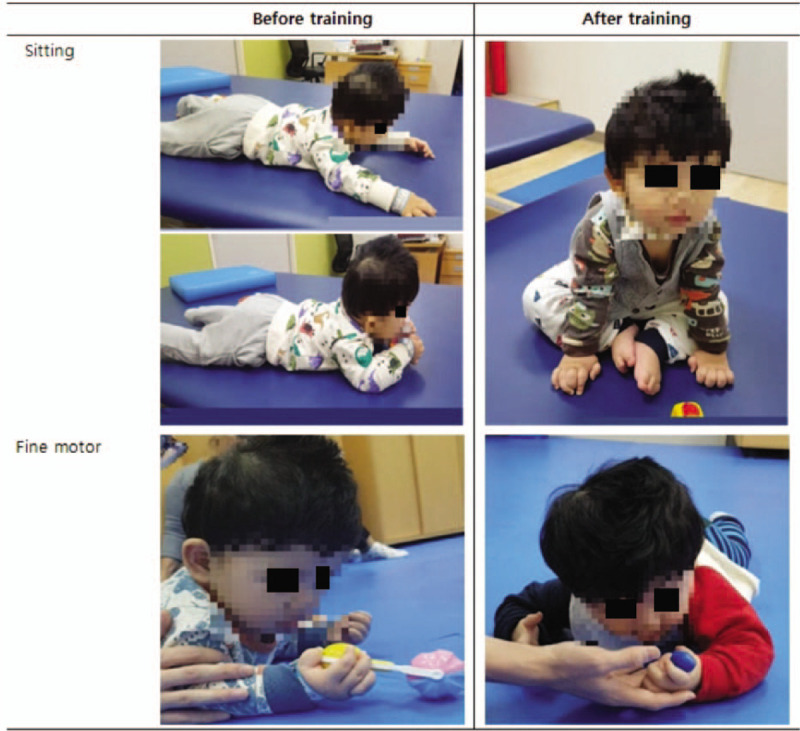
Improvement in gross and fine motor. Before training, the patient could crawl on stomach, while after training he could maintain a sitting position by using his arms (top picture). The patient was able to move objects from one hand to the other before training, and was able to hold objects with his thumb and index finger after training (bottom picture).

The patient was diagnosed with classic PMD, the most common form. According to Tomohiro et al, in classic PMD, development is delayed until 10 years of age and then the patient gradually deteriorates.^[11]^ Our patient received intensive rehabilitation training, including neuro-developmental training to improve balance and developmental delay, during 3 hospitalizations. Before training, the patient could not maintain a sitting position. However, after the first and second training periods, he was able to sit with mild assist. His fine motor function also improved. Before training, he could transfer a cube from one hand to the other, while after training he was able to grab things with his thumb and index finger in a pincher grasp (Fig. 2).

Between the second and third training periods, the patient did not receive any rehabilitation for about 2 months. In the assessment before the third training period, developmental deterioration was observed in the sitting position and with crawling. However, after the third training period, his development improved again (Table 1).

**Table 1:**
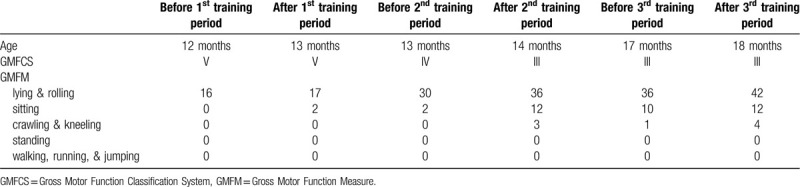
Developmental assessments.

## 4 Discussion

Many studies have examined the treatment of PMD. Mothe and Tator (2008) reported that stem cell transplantation in shiverer mice with demyelinating disease contributed to neural development.^[15]^ Saher et al reported that a cholesterol-enriched diet caused the accumulation of oligodendrocytes in shiverer mice.^[16]^ However, these findings are controversial. Moreover, most of these studies examined mice or monkeys, and not humans. There have been no reported studies of established PMD therapy or treatment protocols, yet.

In general, children with neurologic deficit, such as cerebral palsy, hypoxic or traumatic brain injury, should be evaluated by a comprehensive rehabilitation team as soon as possible after diagnosis. And it is known that rehabilitation treatment should be started early according to the functional state of the patient.^[17–19]^ Reid et al reported that rehabilitation in child with cerebral palsy should begin early. In particular, the first 2 years of life is a significant time for rehabilitation to be more effective than later life.^[20]^ The goal of rehabilitation in child with cerebral palsy is to maximize child's functional abilities, independence and minimize disability. It also prevents secondary complications and improves the quality of life by improving the function of all developmental areas.^[18]^ Unfortunately, no research has been reported on rehabilitation of patients with PMD. Therefore, the initiation timing and functional achievement goal of rehabilitation for PMD was based on the studies of child with cerebral palsy to maximize functional abilities.

We introduced early rehabilitation training for PMD patients. As a result of the rehabilitation program, his balance and development improved. Developmental assessments showed that his gross and fine motor function had improved. Without rehabilitation training, our patients function worsened. However, our patient received no training for about 2 months before the third training admission. The assessments performed at this admission showed that his sitting and crawling ability had deteriorated. Therefore, rehabilitation training contributed to the development of our patient with PMD because developmental deterioration was observed during periods of non-training. Therefore, rehabilitation training can help PMD patients achieve maximal function and catch-up in their growth. In the absence of established PMD therapy, our results suggest that rehabilitation training has few side effects and can be performed at an early stage of the disease.

However, this study examined a single patient and the results cannot be generalized; there has also been no long-term follow-up. Therefore, it is necessary to perform long-term follow-up after a rehabilitation program in patients with PMD. We continue to follow up the patient and will report long-term follow-up functional outcomes of PMD patient after rehabilitation program. This study is valuable as it is the first report on the developmental improvement of a patient with PMD after rehabilitation training.

## Author contributions

**Conceptualization:** Yun-Chol Jang, Bo-ra Mun , Min-Keun Song.

**Funding acquisition:** Min-Keun Song.

**Writing – original draft:** Yun-Chol Jang.

**Writing – review & editing:** Yun-Chol Jang, Min-Keun Song, In Sung Choi.
